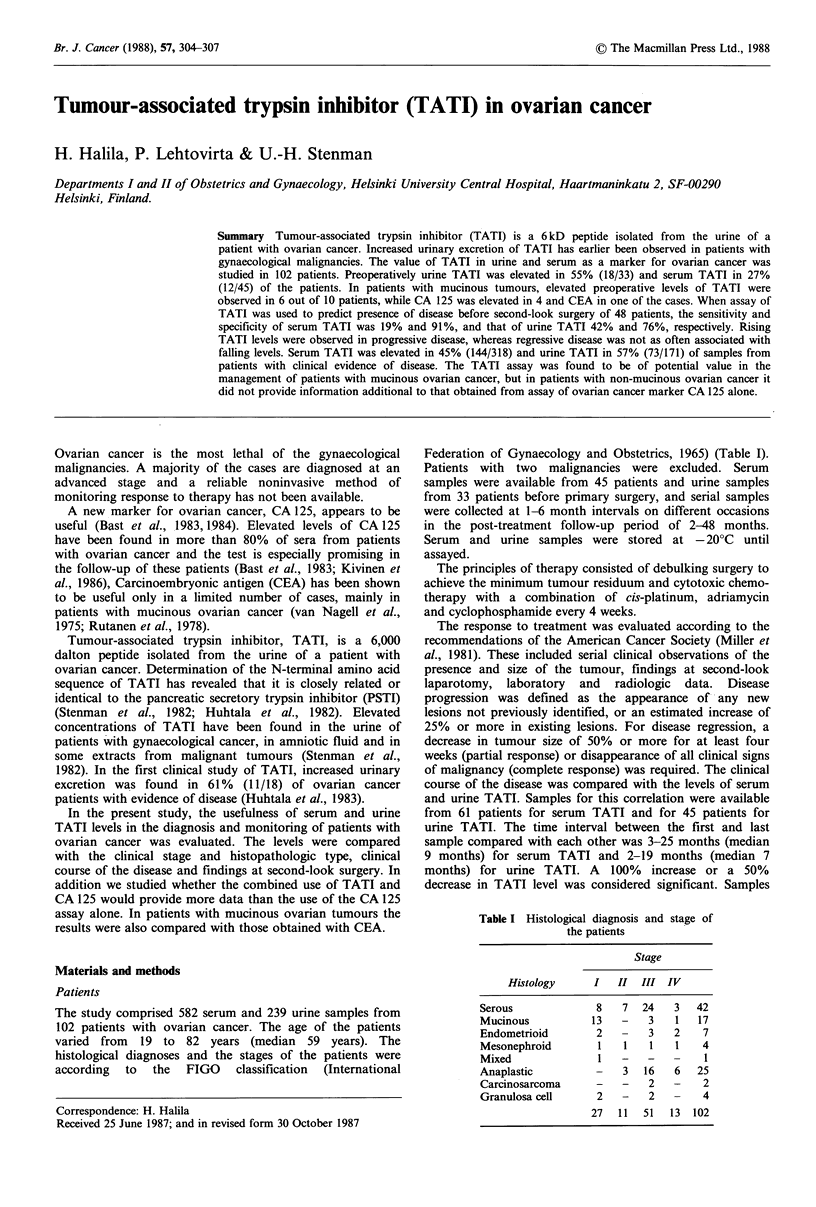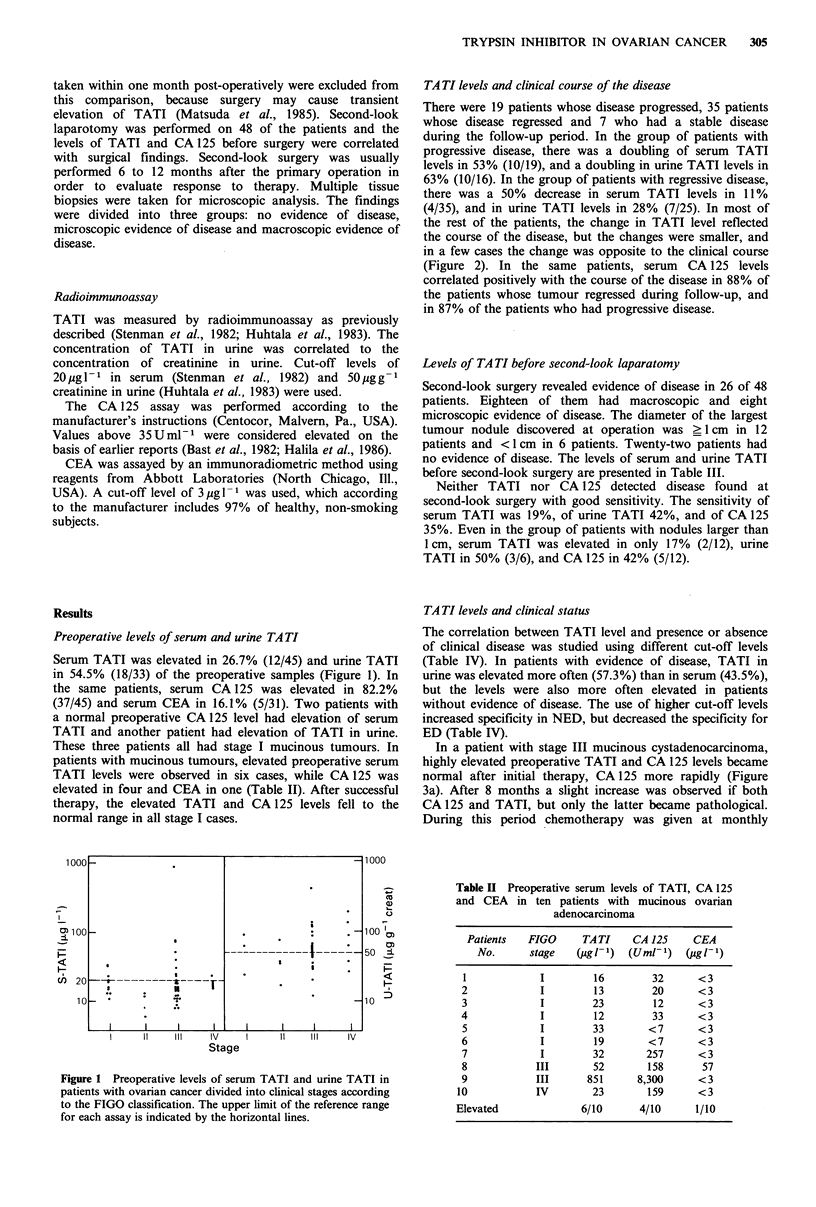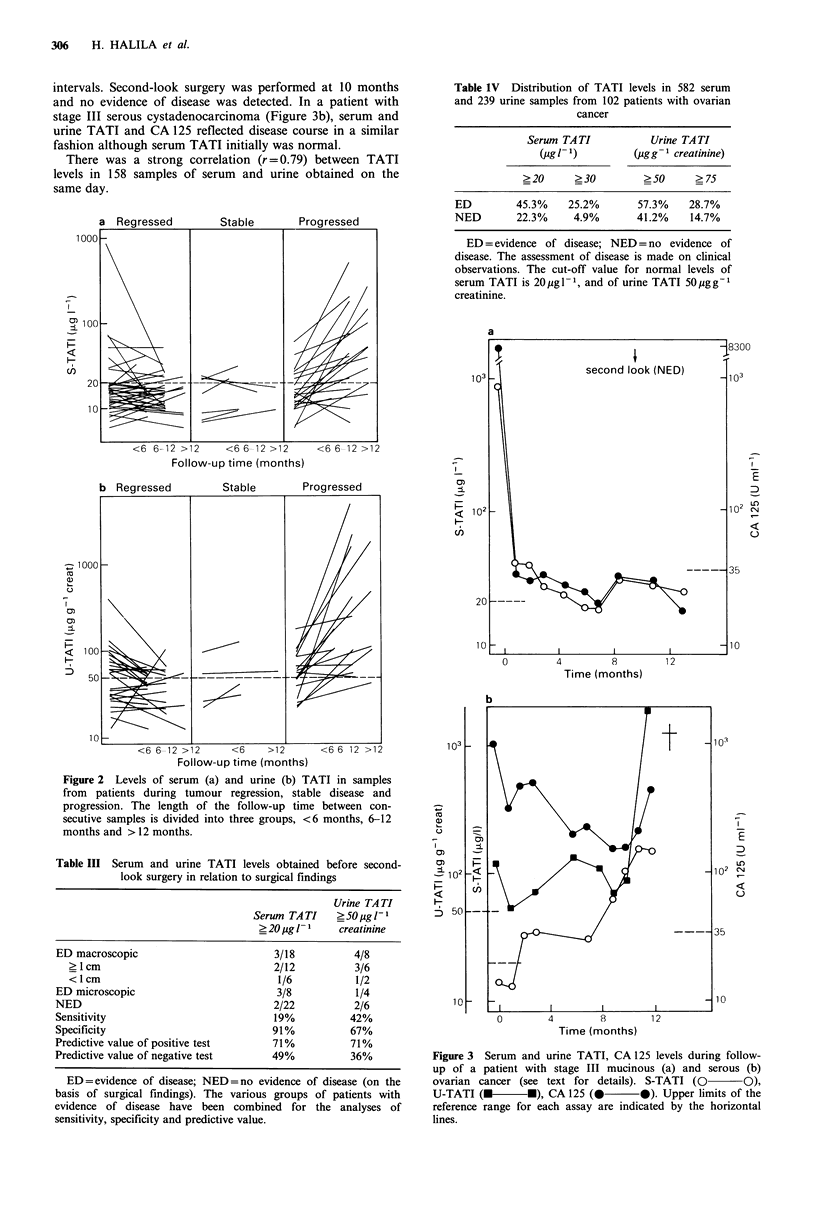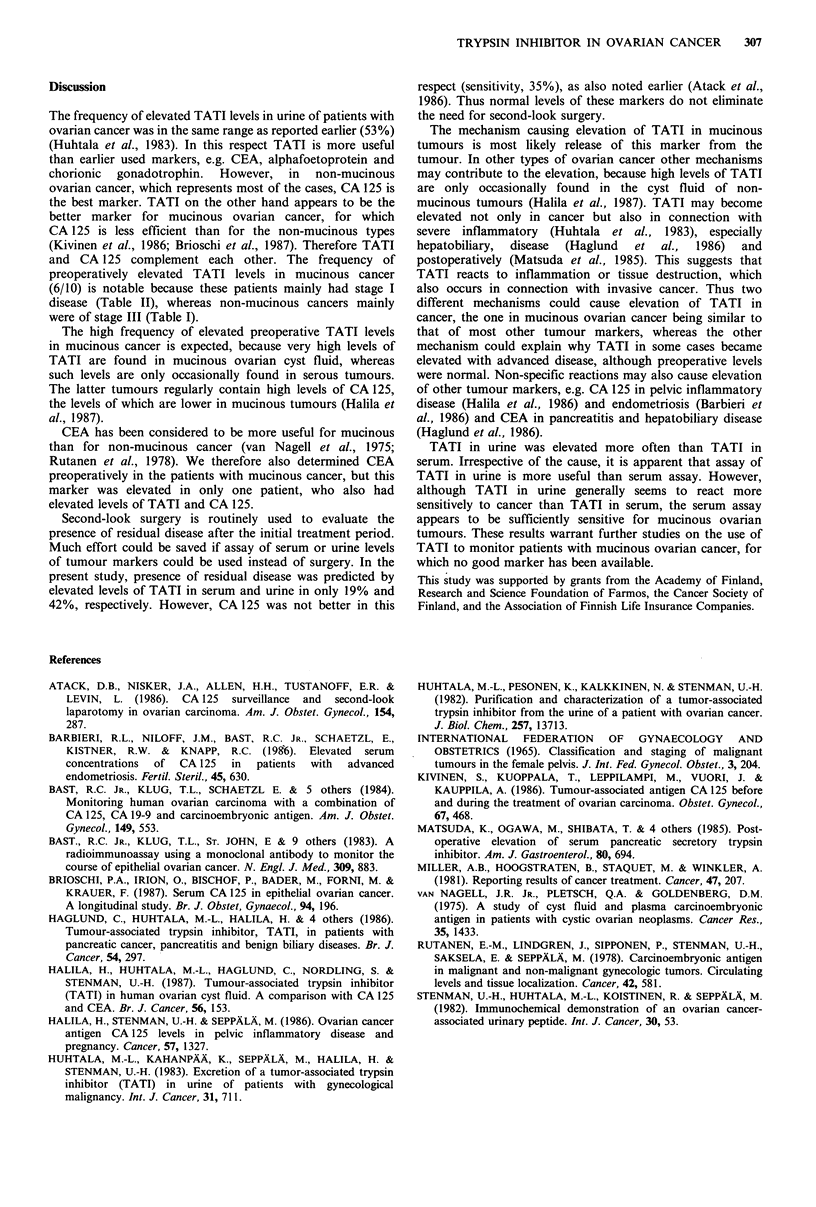# Tumour-associated trypsin inhibitor (TATI) in ovarian cancer.

**DOI:** 10.1038/bjc.1988.67

**Published:** 1988-03

**Authors:** H. Halila, P. Lehtovirta, U. H. Stenman

**Affiliations:** Department I of Obstetrics and Gynaecology, Helsinki University Central Hospital, Finland.

## Abstract

Tumour-associated trypsin inhibitor (TATI) is a 6 kD peptide isolated from the urine of a patient with ovarian cancer. Increased urinary excretion of TATI has earlier been observed in patients with gynaecological malignancies. The value of TATI in urine and serum as a marker for ovarian cancer was studied in 102 patients. Preoperatively urine TATI was elevated in 55% (18/33) and serum TATI in 27% (12/45) of the patients. In patients with mucinous tumours, elevated preoperative levels of TATI were observed in 6 out of 10 patients, while CA 125 was elevated in 4 and CEA in one of the cases. When assay of TATI was used to predict presence of disease before second-look surgery of 48 patients, the sensitivity and specificity of serum TATI was 19% and 91%, and that of urine TATI 42% and 76%, respectively. Rising TATI levels were observed in progressive disease, whereas regressive disease was not as often associated with falling levels. Serum TATI was elevated in 45% (144/318) and urine TATI in 57% (73/171) of samples from patients with clinical evidence of disease. The TATI assay was found to be of potential value in the management of patients with mucinous ovarian cancer, but in patients with non-mucinous ovarian cancer it did not provide information additional to that obtained from assay of ovarian cancer marker CA 125 alone.


*Br. J. Cancer* (1988), **57**, 304–307 © The Macmillan Press Ltd., 1988

### Tumour-associated trypsin inhibitor (TATI) in ovarian cancer

H. Halila, P. Lehtovirta & U.-H. Stenman

*Departments I and II of Obstetrics and Gynaecology, Helsinki University Central Hospital, Haartmaninkatu 2, SF-00290 Helsinki, Finland.*

**Summary** Tumour-associated trypsin inhibitor (TATI) is a 6 kD peptide isolated from the urine of a patient with ovarian cancer. Increased urinary excretion of TATI has earlier been observed in patients with gynaecological malignancies. The value of TATI in urine and serum as a marker for ovarian cancer was studied in 102 patients. Preoperatively urine TATI was elevated in 55% (18/33) and serum TATI in 27% (12/45) of the patients. In patients with mucinous tumours, elevated preoperative levels of TATI were observed in 6 out of 10 patients, while CA 125 was elevated in 4 and CEA in one of the cases. When assay of TATI was used to predict presence of disease before second-look surgery of 48 patients, the sensitivity and specificity of serum TATI was 19% and 91%, and that of urine TATI 42% and 76%, respectively. Rising TATI levels were observed in progressive disease, whereas regressive disease was not as often associated with falling levels. Serum TATI was elevated in 45% (144/318) and urine TATI in 57% (73/171) of samples from patients with clinical evidence of disease. The TATI assay was found to be of potential value in the management of patients with mucinous ovarian cancer, but in patients with non-mucinous ovarian cancer it did not provide information additional to that obtained from assay of ovarian cancer marker CA 125 alone.

Ovarian cancer is the most lethal of the gynaecological malignancies. A majority of the cases are diagnosed at an advanced stage and a reliable noninvasive method of monitoring response to therapy has not been available.

A new marker for ovarian cancer, CA 125, appears to be useful (Bast *et al.*, 1983, 1984). Elevated levels of CA 125 have been found in more than 80% of sera from patients with ovarian cancer and the test is especially promising in the follow-up of these patients (Bast *et al.*, 1983; Kivinen *et al.*, 1986), Carcinoembryonic antigen (CEA) has been shown to be useful only in a limited number of cases, mainly in patients with mucinous ovarian cancer (van Nagell *et al.*, 1975; Rutanen *et al.*, 1978).

Tumour-associated trypsin inhibitor, TATI, is a 6,000 dalton peptide isolated from the urine of a patient with ovarian cancer. Determination of the N-terminal amino acid sequence of TATI has revealed that it is closely related or identical to the pancreatic secretory trypsin inhibitor (PSTI) (Stenman *et al.*, 1982; Huhtala *et al.*, 1982). Elevated concentrations of TATI have been found in the urine of patients with gynaecological cancer, in amniotic fluid and in some extracts from malignant tumours (Stenman *et al.*, 1982). In the first clinical study of TATI, increased urinary excretion was found in 61% (11/18) of ovarian cancer patients with evidence of disease (Huhtala *et al.*, 1983).

In the present study, the usefulness of serum and urine TATI levels in the diagnosis and monitoring of patients with ovarian cancer was evaluated. The levels were compared with the clinical stage and histopathologic type, clinical course of the disease and findings at second-look surgery. In addition we studied whether the combined use of TATI and CA 125 would provide more data than the use of the CA 125 assay alone. In patients with mucinous ovarian tumours the results were also compared with those obtained with CEA.

### Materials and methods

### *Patients*

The study comprised 582 serum and 239 urine samples from 102 patients with ovarian cancer. The age of the patients varied from 19 to 82 years (median 59 years). The histological diagnoses and the stages of the patients were according to the FIGO classification (International Federation of Gynaecology and Obstetrics, 1965) (Table I). Patients with two malignancies were excluded. Serum samples were available from 45 patients and urine samples from 33 patients before primary surgery, and serial samples were collected at 1–6 month intervals on different occasions in the post-treatment follow-up period of 2–48 months. Serum and urine samples were stored at −20°C until assayed.

The principles of therapy consisted of debulking surgery to achieve the minimum tumour residuum and cytotoxic chemotherapy with a combination of *cis*-platinum, adriamycin and cyclophosphamide every 4 weeks.

The response to treatment was evaluated according to the recommendations of the American Cancer Society (Miller *et al.*, 1981). These included serial clinical observations of the presence and size of the tumour, findings at second-look laparotomy, laboratory and radiologic data. Disease progression was defined as the appearance of any new lesions not previously identified, or an estimated increase of 25% or more in existing lesions. For disease regression, a decrease in tumour size of 50% or more for at least four weeks (partial response) or disappearance of all clinical signs of malignancy (complete response) was required. The clinical course of the disease was compared with the levels of serum and urine TATI. Samples for this correlation were available from 61 patients for serum TATI and for 45 patients for urine TATI. The time interval between the first and last sample compared with each other was 3–25 months (median 9 months) for serum TATI and 2–19 months (median 7 months) for urine TATI. A 100% increase or a 50% decrease in TATI level was considered significant. Samples

**Table I** Histological diagnosis and stage of the patients

| | *Stage* | | | | |
|---|---|---|---|---|---|
| *Histology* | *I* | *II* | *III* | *IV* | |
| Serous | 8 | 7 | 24 | 3 | 42 |
| Mucinous | 13 | – | 3 | 1 | 17 |
| Endometrioid | 2 | – | 3 | 2 | 7 |
| Mesonephroid | 1 | 1 | 1 | 1 | 4 |
| Mixed | 1 | – | – | – | 1 |
| Anaplastic | – | 3 | 16 | 6 | 25 |
| Carcinosarcoma | – | – | 2 | – | 2 |
| Granulosa cell | 2 | – | 2 | – | 4 |
| | 27 | 11 | 51 | 13 | 102 |

Correspondence: H. Halila
Received 25 June 1987; and in revised form 30 October 1987

taken within one month post-operatively were excluded from this comparison, because surgery may cause transient elevation of TATI (Matsuda *et al.*, 1985). Second-look laparotomy was performed on 48 of the patients and the levels of TATI and CA 125 before surgery were correlated with surgical findings. Second-look surgery was usually performed 6 to 12 months after the primary operation in order to evaluate response to therapy. Multiple tissue biopsies were taken for microscopic analysis. The findings were divided into three groups: no evidence of disease, microscopic evidence of disease and macroscopic evidence of disease.

*Radioimmunoassay*

TATI was measured by radioimmunoassay as previously described (Stenman *et al.*, 1982; Huhtala *et al.*, 1983). The concentration of TATI in urine was correlated to the concentration of creatinine in urine. Cut-off levels of $20\,\mu g\,l^{-1}$ in serum (Stenman *et al.*, 1982) and $50\,\mu g\,g^{-1}$ creatinine in urine (Huhtala *et al.*, 1983) were used.

The CA 125 assay was performed according to the manufacturer's instructions (Centocor, Malvern, Pa., USA). Values above $35\,U\,ml^{-1}$ were considered elevated on the basis of earlier reports (Bast *et al.*, 1982; Halila *et al.*, 1986).

CEA was assayed by an immunoradiometric method using reagents from Abbott Laboratories (North Chicago, Ill., USA). A cut-off level of $3\,\mu g\,l^{-1}$ was used, which according to the manufacturer includes 97% of healthy, non-smoking subjects.

### Results

*Preoperative levels of serum and urine TATI*

Serum TATI was elevated in 26.7% (12/45) and urine TATI in 54.5% (18/33) of the preoperative samples (Figure 1). In the same patients, serum CA 125 was elevated in 82.2% (37/45) and serum CEA in 16.1% (5/31). Two patients with a normal preoperative CA 125 level had elevation of serum TATI and another patient had elevation of TATI in urine. These three patients all had stage I mucinous tumours. In patients with mucinous tumours, elevated preoperative serum TATI levels were observed in six cases, while CA 125 was elevated in four and CEA in one (Table II). After successful therapy, the elevated TATI and CA 125 levels fell to the normal range in all stage I cases.

**Figure 1** Preoperative levels of serum TATI and urine TATI in patients with ovarian cancer divided into clinical stages according to the FIGO classification. The upper limit of the reference range for each assay is indicated by the horizontal lines.

*TATI levels and clinical course of the disease*

There were 19 patients whose disease progressed, 35 patients whose disease regressed and 7 who had a stable disease during the follow-up period. In the group of patients with progressive disease, there was a doubling of serum TATI levels in 53% (10/19), and a doubling in urine TATI levels in 63% (10/16). In the group of patients with regressive disease, there was a 50% decrease in serum TATI levels in 11% (4/35), and in urine TATI levels in 28% (7/25). In most of the rest of the patients, the change in TATI level reflected the course of the disease, but the changes were smaller, and in a few cases the change was opposite to the clinical course (Figure 2). In the same patients, serum CA 125 levels correlated positively with the course of the disease in 88% of the patients whose tumour regressed during follow-up, and in 87% of the patients who had progressive disease.

*Levels of TATI before second-look laparatomy*

Second-look surgery revealed evidence of disease in 26 of 48 patients. Eighteen of them had macroscopic and eight microscopic evidence of disease. The diameter of the largest tumour nodule discovered at operation was ≥1 cm in 12 patients and <1 cm in 6 patients. Twenty-two patients had no evidence of disease. The levels of serum and urine TATI before second-look surgery are presented in Table III.

Neither TATI nor CA 125 detected disease found at second-look surgery with good sensitivity. The sensitivity of serum TATI was 19%, of urine TATI 42%, and of CA 125 35%. Even in the group of patients with nodules larger than 1 cm, serum TATI was elevated in only 17% (2/12), urine TATI in 50% (3/6), and CA 125 in 42% (5/12).

*TATI levels and clinical status*

The correlation between TATI level and presence or absence of clinical disease was studied using different cut-off levels (Table IV). In patients with evidence of disease, TATI in urine was elevated more often (57.3%) than in serum (43.5%), but the levels were also more often elevated in patients without evidence of disease. The use of higher cut-off levels increased specificity in NED, but decreased the specificity for ED (Table IV).

In a patient with stage III mucinous cystadenocarcinoma, highly elevated preoperative TATI and CA 125 levels became normal after initial therapy, CA 125 more rapidly (Figure 3a). After 8 months a slight increase was observed if both CA 125 and TATI, but only the latter became pathological. During this period chemotherapy was given at monthly

**Table II** Preoperative serum levels of TATI, CA 125 and CEA in ten patients with mucinous ovarian adenocarcinoma

| *Patients No.* | *FIGO stage* | *TATI* ($\mu g\,l^{-1}$) | *CA 125* ($U\,ml^{-1}$) | *CEA* ($\mu g\,l^{-1}$) |
|---|---|---|---|---|
| 1 | I | 16 | 32 | <3 |
| 2 | I | 13 | 20 | <3 |
| 3 | I | 23 | 12 | <3 |
| 4 | I | 12 | 33 | <3 |
| 5 | I | 33 | <7 | <3 |
| 6 | I | 19 | <7 | <3 |
| 7 | I | 32 | 257 | <3 |
| 8 | III | 52 | 158 | 57 |
| 9 | III | 851 | 8,300 | <3 |
| 10 | IV | 23 | 159 | <3 |
| Elevated | | 6/10 | 4/10 | 1/10 |

intervals. Second-look surgery was performed at 10 months and no evidence of disease was detected. In a patient with stage III serous cystadenocarcinoma (Figure 3b), serum and urine TATI and CA 125 reflected disease course in a similar fashion although serum TATI initially was normal.

There was a strong correlation ($r = 0.79$) between TATI levels in 158 samples of serum and urine obtained on the same day.

**Figure 2** Levels of serum (a) and urine (b) TATI in samples from patients during tumour regression, stable disease and progression. The length of the follow-up time between consecutive samples is divided into three groups, <6 months, 6–12 months and >12 months.

**Table III** Serum and urine TATI levels obtained before second-look surgery in relation to surgical findings

| | *Serum TATI* $\geq 20\,\mu g\,l^{-1}$ | *Urine TATI* $\geq 50\,\mu g\,l^{-1}$ *creatinine* |
|---|---|---|
| ED macroscopic | 3/18 | 4/8 |
| ≧1 cm | 2/12 | 3/6 |
| <1 cm | 1/6 | 1/2 |
| ED microscopic | 3/8 | 1/4 |
| NED | 2/22 | 2/6 |
| Sensitivity | 19% | 42% |
| Specificity | 91% | 67% |
| Predictive value of positive test | 71% | 71% |
| Predictive value of negative test | 49% | 36% |

ED = evidence of disease; NED = no evidence of disease (on the basis of surgical findings). The various groups of patients with evidence of disease have been combined for the analyses of sensitivity, specificity and predictive value.

**Table IV** Distribution of TATI levels in 582 serum and 239 urine samples from 102 patients with ovarian cancer

| | *Serum TATI* ($\mu g\,l^{-1}$) | | *Urine TATI* ($\mu g\,g^{-1}$ *creatinine*) | |
|---|---|---|---|---|
| | ≧20 | ≧30 | ≧50 | ≧75 |
| ED | 45.3% | 25.2% | 57.3% | 28.7% |
| NED | 22.3% | 4.9% | 41.2% | 14.7% |

ED = evidence of disease; NED = no evidence of disease. The assessment of disease is made on clinical observations. The cut-off value for normal levels of serum TATI is $20\,\mu g\,l^{-1}$, and of urine TATI $50\,\mu g\,g^{-1}$ creatinine.

**Figure 3** Serum and urine TATI, CA 125 levels during follow-up of a patient with stage III mucinous (a) and serous (b) ovarian cancer (see text for details). S-TATI (○——○), U-TATI (■——■), CA 125 (●——●). Upper limits of the reference range for each assay are indicated by the horizontal lines.

### Discussion

The frequency of elevated TATI levels in urine of patients with ovarian cancer was in the same range as reported earlier (53%) (Huhtala *et al.*, 1983). In this respect TATI is more useful than earlier used markers, e.g. CEA, alphafoetoprotein and chorionic gonadotrophin. However, in non-mucinous ovarian cancer, which represents most of the cases, CA 125 is the best marker. TATI on the other hand appears to be the better marker for mucinous ovarian cancer, for which CA 125 is less efficient than for the non-mucinous types (Kivinen *et al.*, 1986; Brioschi *et al.*, 1987). Therefore TATI and CA 125 complement each other. The frequency of preoperatively elevated TATI levels in mucinous cancer (6/10) is notable because these patients mainly had stage I disease (Table II), whereas non-mucinous cancers mainly were of stage III (Table I).

The high frequency of elevated preoperative TATI levels in mucinous cancer is expected, because very high levels of TATI are found in mucinous ovarian cyst fluid, whereas such levels are only occasionally found in serous tumours. The latter tumours regularly contain high levels of CA 125, the levels of which are lower in mucinous tumours (Halila *et al.*, 1987).

CEA has been considered to be more useful for mucinous than for non-mucinous cancer (van Nagell *et al.*, 1975; Rutanen *et al.*, 1978). We therefore also determined CEA preoperatively in the patients with mucinous cancer, but this marker was elevated in only one patient, who also had elevated levels of TATI and CA 125.

Second-look surgery is routinely used to evaluate the presence of residual disease after the initial treatment period. Much effort could be saved if assay of serum or urine levels of tumour markers could be used instead of surgery. In the present study, presence of residual disease was predicted by elevated levels of TATI in serum and urine in only 19% and 42%, respectively. However, CA 125 was not better in this respect (sensitivity, 35%), as also noted earlier (Atack *et al.*, 1986). Thus normal levels of these markers do not eliminate the need for second-look surgery.

The mechanism causing elevation of TATI in mucinous tumours is most likely release of this marker from the tumour. In other types of ovarian cancer other mechanisms may contribute to the elevation, because high levels of TATI are only occasionally found in the cyst fluid of non-mucinous tumours (Halila *et al.*, 1987). TATI may become elevated not only in cancer but also in connection with severe inflammatory (Huhtala *et al.*, 1983), especially hepatobiliary, disease (Haglund *et al.*, 1986) and postoperatively (Matsuda *et al.*, 1985). This suggests that TATI reacts to inflammation or tissue destruction, which also occurs in connection with invasive cancer. Thus two different mechanisms could cause elevation of TATI in cancer, the one in mucinous ovarian cancer being similar to that of most other tumour markers, whereas the other mechanism could explain why TATI in some cases became elevated with advanced disease, although preoperative levels were normal. Non-specific reactions may also cause elevation of other tumour markers, e.g. CA 125 in pelvic inflammatory disease (Halila *et al.*, 1986) and endometriosis (Barbieri *et al.*, 1986) and CEA in pancreatitis and hepatobiliary disease (Haglund *et al.*, 1986).

TATI in urine was elevated more often than TATI in serum. Irrespective of the cause, it is apparent that assay of TATI in urine is more useful than serum assay. However, although TATI in urine generally seems to react more sensitively to cancer than TATI in serum, the serum assay appears to be sufficiently sensitive for mucinous ovarian tumours. These results warrant further studies on the use of TATI to monitor patients with mucinous ovarian cancer, for which no good marker has been available.

This study was supported by grants from the Academy of Finland, Research and Science Foundation of Farmos, the Cancer Society of Finland, and the Association of Finnish Life Insurance Companies.

### References

ATACK, D.B., NISKER, J.A., ALLEN, H.H., TUSTANOFF, E.R. & LEVIN, L. (1986). CA 125 surveillance and second-look laparotomy in ovarian carcinoma. *Am. J. Obstet. Gynecol.*, **154**, 287.

BARBIERI, R.L., NILOFF, J.M., BAST, R.C. Jr., SCHAETZL, E., KISTNER, R.W. & KNAPP, R.C. (1986). Elevated serum concentrations of CA 125 in patients with advanced endometriosis. *Fertil. Steril.*, **45**, 630.

BAST, R.C. Jr., KLUG, T.L., SCHAETZL E. & 5 others (1984). Monitoring human ovarian carcinoma with a combination of CA 125, CA 19-9 and carcinoembryonic antigen. *Am. J. Obstet. Gynecol.*, **149**, 553.

BAST., R.C. Jr., KLUG, T.L., St. JOHN, E & 9 others (1983). A radioimmunoassay using a monoclonal antibody to monitor the course of epithelial ovarian cancer. *N. Engl. J. Med.*, **309**, 883.

BRIOSCHI, P.A., IRION, O., BISCHOF, P., BADER, M., FORNI, M. & KRAUER, F. (1987). Serum CA 125 in epithelial ovarian cancer. A longitudinal study. *Br. J. Obstet, Gynaecol.*, **94**, 196.

HAGLUND, C., HUHTALA, M.-L., HALILA, H. & 4 others (1986). Tumour-associated trypsin inhibitor, TATI, in patients with pancreatic cancer, pancreatitis and benign biliary diseases. *Br. J. Cancer*, **54**, 297.

HALILA, H., HUHTALA, M.-L., HAGLUND, C., NORDLING, S. & STENMAN, U.-H. (1987). Tumour-associated trypsin inhibitor (TATI) in human ovarian cyst fluid. A comparison with CA 125 and CEA. *Br. J. Cancer*, **56**, 153.

HALILA, H., STENMAN, U.-H. & SEPPÄLÄ, M. (1986). Ovarian cancer antigen CA 125 levels in pelvic inflammatory disease and pregnancy. *Cancer*, **57**, 1327.

HUHTALA, M.-L., KAHANPÄÄ, K., SEPPÄLÄ, M., HALILA, H. & STENMAN, U.-H. (1983). Excretion of a tumor-associated trypsin inhibitor (TATI) in urine of patients with gynecological malignancy. *Int. J. Cancer*, **31**, 711.

HUHTALA, M.-L., PESONEN, K., KALKKINEN, N. & STENMAN, U.-H. (1982). Purification and characterization of a tumor-associated trypsin inhibitor from the urine of a patient with ovarian cancer. *J. Biol. Chem.*, **257**, 13713.

INTERNATIONAL FEDERATION OF GYNAECOLOGY AND OBSTETRICS (1965). Classification and staging of malignant tumours in the female pelvis. *J. Int. Fed. Gynecol. Obstet.*, **3**, 204.

KIVINEN, S., KUOPPALA, T., LEPPILAMPI, M., VUORI, J. & KAUPPILA, A. (1986). Tumour-associated antigen CA 125 before and during the treatment of ovarian carcinoma. *Obstet. Gynecol.*, **67**, 468.

MATSUDA, K., OGAWA, M., SHIBATA, T. & 4 others (1985). Post-operative elevation of serum pancreatic secretory trypsin inhibitor. *Am. J. Gastroenterol.*, **80**, 694.

MILLER, A.B., HOOGSTRATEN, B., STAQUET, M. & WINKLER, A. (1981). Reporting results of cancer treatment. *Cancer*, **47**, 207.

van NAGELL, J.R. Jr., PLETSCH, Q.A. & GOLDENBERG, D.M. (1975). A study of cyst fluid and plasma carcinoembryonic antigen in patients with cystic ovarian neoplasms. *Cancer Res.*, **35**, 1433.

RUTANEN, E.-M., LINDGREN, J., SIPPONEN, P., STENMAN, U.-H., SAKSELA, E. & SEPPÄLÄ, M. (1978). Carcinoembryonic antigen in malignant and non-malignant gynecologic tumors. Circulating levels and tissue localization. *Cancer*, **42**, 581.

STENMAN, U.-H., HUHTALA, M.-L., KOISTINEN, R. & SEPPÄLÄ, M. (1982). Immunochemical demonstration of an ovarian cancer-associated urinary peptide. *Int. J. Cancer*, **30**, 53.